# Diverse integrin adhesion stoichiometries caused by varied actomyosin activity

**DOI:** 10.1098/rsob.160250

**Published:** 2017-04-26

**Authors:** Natalia A. Bulgakova, Jutta Wellmann, Nicholas H. Brown

**Affiliations:** Department of Physiology, Development and Neuroscience and The Gurdon Institute, University of Cambridge, Downing Street, Cambridge CB2 3DY, UK

**Keywords:** integrin, contractility, *Drosophila*, muscle, myosin, stoichiometry

## Abstract

Cells in an organism are subjected to numerous sources of external and internal forces, and are able to sense and respond to these forces. Integrin-mediated adhesion links the extracellular matrix outside cells to the cytoskeleton inside, and participates in sensing, transmitting and responding to forces. While integrin adhesion rapidly adapts to changes in forces in isolated migrating cells, it is not known whether similar or more complex responses occur within intact, developing tissues. Here, we studied changes in integrin adhesion composition upon different contractility conditions in *Drosophila* embryonic muscles. We discovered that all integrin adhesion components tested were still present at muscle attachment sites (MASs) when either cytoplasmic or muscle myosin II was genetically removed, suggesting a primary role of a developmental programme in the initial assembly of integrin adhesions. Contractility does, however, increase the levels of integrin adhesion components, suggesting a mechanism to balance the strength of muscle attachment to the force of muscle contraction. Perturbing contractility in distinct ways, by genetic removal of either cytoplasmic or muscle myosin II or eliminating muscle innervation, each caused unique alterations to the stoichiometry at MASs. This suggests that different integrin-associated proteins are added to counteract different kinds of force increase.

## 1. Introduction

All cells in an organism are exposed to numerous sources of mechanical forces. Some forces originate from the external environment, including compression, tension and shear stress. Others are generated within cells by actomyosin activity and osmotic pressure. In turn, cells sense applied forces and respond accordingly, by changing their shape or position within an organism, as well as dividing or differentiating [1,2]. The integrated response of cells to different forces is crucial for morphogenesis of tissues and their ability to withstand applied forces, and its failure is implicated in various diseases, including atherosclerosis and muscular dystrophy (reviewed in [3,4]).

Integrins are heterodimeric transmembrane receptors that link the extracellular matrix (ECM) to the cytoskeleton and signalling pathways within cells. This link is mediated by a large set of intracellular integrin-associated proteins (IAPs), which interact either with the intracellular domains of integrins or with each other [5]. Consistent with the function of integrins in linking the cell's exterior environment to the interior cytoskeleton, integrin–ECM adhesion participates in force transmission, sensing and responding to a wide range of forces (reviewed in [6]). Any force, whether generated inside or outside the cell, is balanced by a counter-force, and integrins mediate force transmission in either direction. New integrin adhesion sites form where the force is applied to cells externally [7,8], and conversely, blocking intracellular forces by inhibition of actomyosin contractility causes a rapid disassembly of integrin adhesion sites [9]. Force enhances recruitment of IAPs such as talin, vinculin and paxillin [8,10–13]. One mechanism for force-dependent recruitment is the unfolding of IAP domains to expose new binding sites; for example, force on talin reveals cryptic vinculin-binding sites [14] and, reciprocally, force on vinculin stabilizes its association with the adhesion site [15], possibly by stabilizing its binding to talin and actin.

While rapid, force-dependent changes to integrin adhesion are important in migrating cells, it is not clear how well this paradigm translates to integrin adhesion sites within intact tissues, both during the formation of the tissues during development and in their maintenance throughout life. A key function of integrins during development is to mediate adhesion between different cell layers, via an intervening ECM [16,17]. For example, in the *Drosophila* embryo, the highest levels of integrins are found at muscle ends, where muscles attach to each other and the tendon cells in the overlying epidermis, via the tendon matrix, to form muscle attachment sites (MASs) [18]. In the absence of integrins, once the muscles begin contracting, they detach and round up. Therefore, it seems likely that some level of integrin-mediated adhesion must develop prior to the strong forces that arise as the contractile apparatus is formed.

In this report, we examined how forces provided by actomyosin contractility affect recruitment of integrin and IAPs *in vivo* during development. We used *Drosophila* embryonic MASs as a model, as they are well characterized, accessible for live imaging and have highly reproducible levels of IAP accumulation from muscle to muscle, and from animal to animal ([16], this work). Contraction of the actomyosin cytoskeleton generates tension on MASs. Two type II myosins are present in muscle cells: the ubiquitously expressed cytoplasmic myosin II, which is enriched at the MASs and Z-lines, and muscle myosin, which is the main constituent of sarcomeres [19,20]. We used null mutations in the genes encoding the heavy chain of each of the two myosin IIs (*zipper* (*zip*) and *Myosin Heavy Chain* (*Mhc*), respectively) to reduce intracellular contractility within muscle cells.

The ability of the muscles to contract arises progressively during development. The first contractions occur as the sarcomeres start assembling and comprise brief twitches of individual muscles. As the synapses between motor axons and muscles develop, neuronal activity induces short periodic bursts of increased muscle contraction (bursting activity), which involve multiple muscles on both sides of the embryo. The muscle contractions finally mature to complete sequences of forward and backward waves of peristaltic contractility by the end of embryogenesis [21]. The periodic bursts are separated by quieter inter-bursting periods, when only isolated contractions of individual muscles occur. To block bursting activity but not the isolated inter-bursting contractions, we blocked neuronal innervation with a null mutation in the gene encoding the muscle-specific subunit of the glutamate receptor (*GluRIIC*) [21].

We monitored the effects of these genetic force perturbations on the assembly of integrin adhesion complexes using fluorescently tagged integrins and a selection of eight IAPs (talin, integrin-linked kinase (ILK), PINCH, tensin, fermitin1 (fit1; a kindlin orthologue), GIT, paxillin and vinculin), all expressed with their own promoter at endogenous levels. These IAPs were selected because they are all recruited to MASs, they vary in the importance of their function at the MASs, ranging from absolutely required (talin) to dispensable (vinculin) (for references, see [17]) and they represent different subcomplexes within integrin adhesions [22,23]. It is possible that all IAPs are mechanotransducers, i.e. make protein interactions that are regulated by force, but to date this is only well documented for talin and vinculin.

We found that all nine proteins accumulated less when force was reduced in different ways, but their responses were surprisingly diverse. There was not a simple correlation between the reduction in muscle contraction and reduction in IAP levels, and thus the response to changes in muscle contraction is nonlinear. Altogether, our findings demonstrate a complex relationship between the composition of integrin adhesions and the contractile forces within muscles.

## 2. Results

### 2.1. Muscle contractility is differently reduced in *Mhc^1^*, *GluRIIC^1^* or *zip^2^* mutants

**Figure 1.**
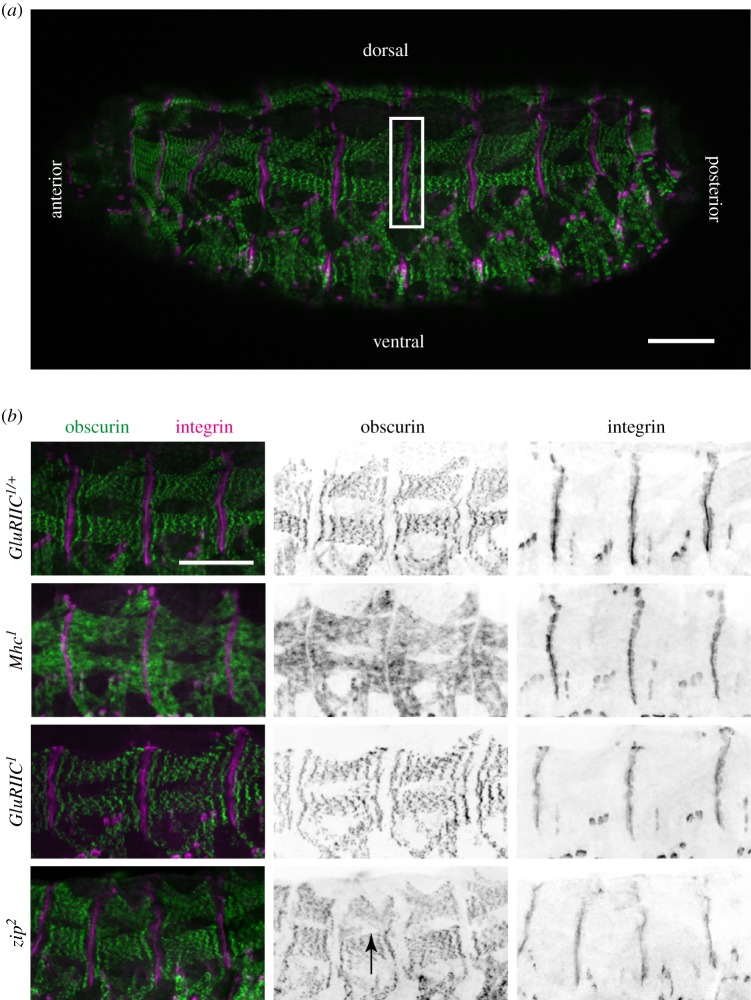
Embryos homozygous for *Mhc^1^*, *GluRIIC^1^* or *zip^2^* have normal muscle morphology. (*a*) Overview of muscle pattern in a control embryo (*GluRIIC^1^*/+) with indicated anterior, posterior, dorsal and ventral sides. White rectangle indicates the extent of the dorsal MAS. (*b*) Close-up view on dorsal muscles in *GluRIIC^1^*/+, *Mhc^1^*, *GluRIIC^1^* and *zip^2^* embryos. Black arrow demonstrates a mild defect in muscle morphology in proximity to a dorsal hole. All embryos were stained with anti-obscurin (green and middle column in (*b*)), anti-αPS2 (magenta, right column in (*b*)) and anti-GFP (not shown) to distinguish between homozygote and heterozygote embryos. Scale bar, 50 µm.

To test the role of muscle contractility on the accumulation of integrins and IAPs at MASs, we focused on embryos 19–20 h after egg laying, close to the end of embryogenesis and therefore just before hatching, as by this time the embryos develop a complete pattern of coordinated contractility [21]. All measurements were performed on the same MASs, which are formed by the dorsal muscles (figure 1*a*). These attachments are formed by the adhesion of the four dorsal muscles in each hemi-segment to the tendon cells in the overlying epidermis and end-to-end adhesion to the dorsal muscles in adjacent segments. Depending on the focal plane, this MAS appears as a series of attachment sites formed by individual tendon cells, when viewed more superficially, or as a continuous line when viewed more deeply, at the level of the muscles (see electronic supplementary material, movie S1). The dorsal muscle attachments were selected because they are easy to identify in the images and formed by a small number of muscles. (For a diagram of muscles, see [24]).

First, we tested whether removing muscle myosin (*Mhc)*, neuronal innervation (*GluRIIC*) or cytoplasmic myosin II (*zip)* had a strong effect on overall muscle morphology and sarcomeric structure (figure 1*b*). The overall shape of the dorsal muscles appeared normal in all three mutants. As expected, the sarcomeric structure was completely disrupted in the absence of muscle myosin, as visualized by examining a robust marker of sarcomeric structure: antibody staining of the M-band protein obscurin (Unc-89) [25]. Obscurin distribution in *GluRIIC^1^* homozygotes was indistinguishable from the control. In embryos lacking cytoplasmic myosin II, the sarcomeric structure was intact, but the muscles were 33% shorter (*p* < 0.0001), which appeared to be caused by the fully penetrant defects in head involution, and the muscles also occasionally showed morphological defects (arrow in figure 1*b*) that correlated with the more variable defects in dorsal closure. Previous work showed that cytoplasmic myosin II is enriched at MASs and that the actin fails to become well organized in sarcomeres in *zip* mutant embryos [19], showing that there is some disruption to sarcomere formation, but the presence of M-lines is consistent with the ability of muscles lacking cytoplasmic myosin to contract (as documented below). It should also be mentioned that cytoplasmic myosin II is deposited maternally into the egg, and is essential for earlier developmental events, such as cellularization [26]. The morphogenetic defects that occur at later stages in embryos that are unable to make any new myosin II (*zip^2^* homozygotes) demonstrates that the maternally deposited protein is not sufficient for later developmental events, and at the stage examined here we could no longer detect maternally provided myosin II tagged with YFP (N.A.B. and N.H.B. 2012, our unpublished observations). As expected, there is no maternally provided muscle myosin mRNA [27].

**Figure 2.**
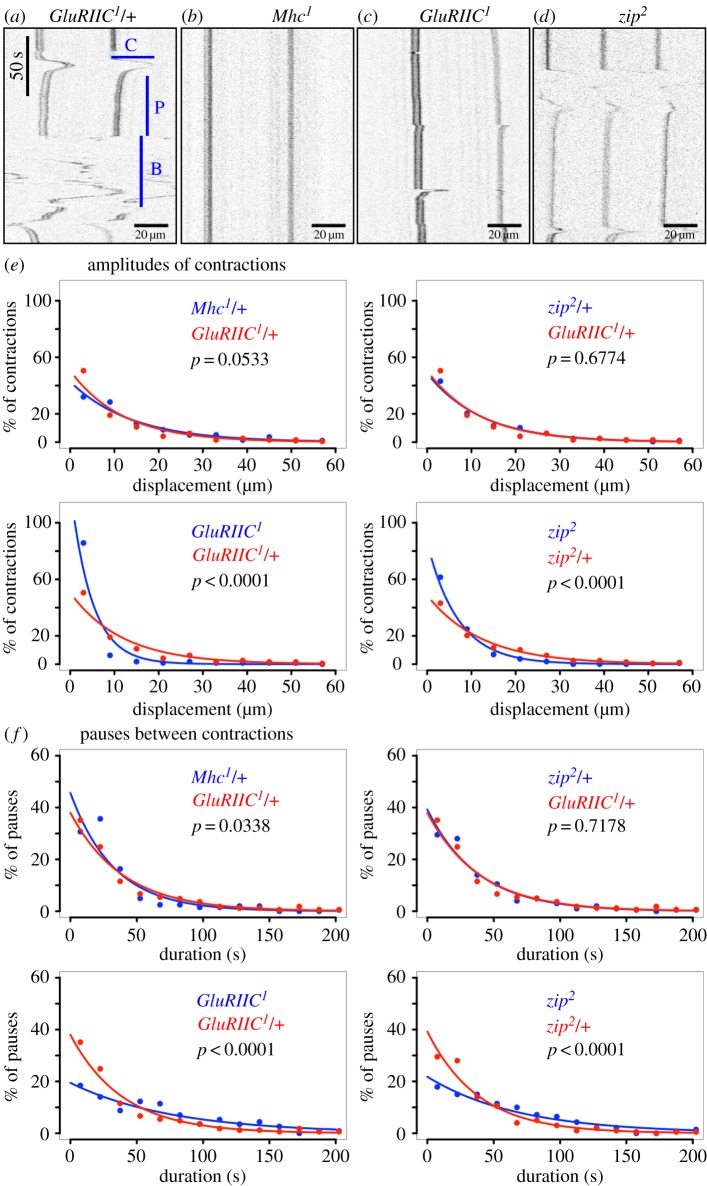
Characterization of muscle contractility in embryos heterozygous and homozygous for *Mhc^1^*, *GluRIIC^1^* or *zip^2^*. (*a*–*d*) Examples of kymographs obtained from *in vivo* imaging of *GluRIIC^1^*/+ (*a*), *Mhc^1^* (*b*), *GluRIIC^1^* (*c*) and *zip^2^* (*d*) embryos, each expressing a paternal copy of GFP-talin. Examples of inter-bursting individual contraction (C), pause between contractions (P) and bursting activity (B) are demonstrated in (*a*). (*e*,*f*) Distributions of amplitudes of contractions (*e*) and pauses between contractions (*f*). The dots represent the percentage of contractions with a particular binned amplitude (*e*) and pauses with a particular binned duration (*f*). The best fit curves are shown in solid lines. The *p*-values correspond to comparison between two distributions depicted in each graph. The exact values of contractility parameters, sample sizes and *p*-values are in table 1.

Most muscle contractions during late embryogenesis occur within periods of bursting activity (electronic supplementary material, movie S1, B in figure 2*a*); thus, we quantified how the absence of cytoplasmic myosin II (*zip),* muscle myosin II (*Mhc)* and neuronal innervation (*GluRIIC*) affected the bursts. The durations of bursts varied up to 20-fold in controls, making it an unreliable measure. Instead, we used the average interval between bursts, which did not differ between control embryos heterozygous for *Mhc^1^*, *GluRIIC^1^* or *zip^2^* (table 1; see Material and methods). As expected, *Mhc^1^* and *GluRIIC^1^* homozygous embryos completely lacked bursting activity (electronic supplementary material, movies S2 and S3; figure 2*b,c*). By contrast, the bursting activity was present in *zip^2^* homozygous embryos (electronic supplementary material, movie S4; figure 2*d*), but bursts occurred less often as reflected by an increased average interval (table 1).

**Table 1.** Muscle contractility in embryos heterozygous and homozygous for *Mhc^1^*, *GluRIIC^1^* and *zip^2^*. Effects of *Mhc^1^*, *GluRIIC^1^* and *zip^2^* mutations on muscle contractility. n.i., not identified.

genotype (number of analysed embryos)	median time between bursts of contraction (min, mean ± s.d.)	*p*-value (compared with)	median amplitude of contraction waves (µm, mean ± s.d.)	*p*-value (compared with)	median duration of pauses (s, mean ± s.d.)	*p*-value (compared with)
*Mhc^1^/+* (22)	7.6 ± 1.6	0.5601 (*GluRIIC^1^/+*)	9.8 ± 0.6*	0.0541 (*GluRIIC^1^/+*)	22.9 ± 1.6*	0.0337 (*GluRIIC^1^/+*)
*GluRIIC^1^/+*(21)	9.0 ± 2.1		8.3 ± 0.6		27.5 ± 2.2	
*zip^2^/+* (20)	13.2 ± 4.0	0.2817 (*GluRIIC^1^/+*)	8.5 ± 0.5	0.7107 (*GluRIIC^1^/+*)	26.6 ± 1.9	0.7210 (*GluRIIC^1^/+*)
*GluRIIC^1^* (18)	n.i.		3.3 ± 0.3***	<0.0001 (*GluRIIC^1^/+*)	53.5 ± 5.1***	<0.0001 (*GluRIIC^1^/+*)
*zip^2^* (21)	36.0 ± 20.1*^,#^	0.0428 (*GluRIIC^1^/+*) 0.0131 (*GluRIIC^1^*)	4.8 ± 0.4***^,##^	<0.0001 (*GluRIIC^1^/+*) 0.0011 (*GluRIIC^1^*)	47.6 ± 4.1***	<0.0001 (*GluRIIC^1^/+*) 0.3094 (*GluRIIC^1^*)

*p < 0.1 and ***p < 0.0001 for comparisons with heterozygous controls; ^#^*p* < 0.1; ^##^*p* < 0.01 for comparisons between mutants.

During intervals between bursts of contractility, there is an additional source of contractile force as individual muscles occasionally contract [21]. We quantified how *zip^2^, Mhc^1^* and *GluRIIC^1^* mutations affected the individual contractions during inter-bursting intervals using two measures (see Material and methods): the amplitudes of individual contractions (C in figure 2*a*), i.e. the distance of MAS displacement during each contraction, and the duration of the pauses between sequential contractions (P in figure 2*a*). Distributions of amplitudes and pauses were best fit by exponential distributions (figure 2*e*,*f*), suggesting that both are Poisson processes, indicating that individual contraction events occur continuously and independently of each other. Homozygosity for *Mhc^1^* completely abolished any contractility (electronic supplementary material, movie S2; figure 2*b*), and therefore we did not measure contraction amplitudes and pauses. By contrast, heterozygosity for *Mhc^1^* produced a mild dominant phenotype with slightly more frequent contractions with higher amplitudes in comparison to *GluRIIC^1^* and *zip^2^* heterozygotes, which did not differ from each other (figure 2*e*,*f* and table 1). This is consistent with the dominant flightless phenotype of this mutant [28]. In *zip^2^* and *GluRIIC^1^* homozygous embryos, the individual contractions had lower amplitudes followed by longer pauses than in heterozygous siblings (figure 2*e*,*f* and table 1). Thus, the three selected mutants reduced muscle contractility to different extents: *Mhc^1^* completely abolished any contractility; *GluRIIC^1^* abolished bursting activity, and reduced inter-bursting contractility; *zip^2^* maintained but reduced both bursting and inter-bursting contractility.

### 2.2. Adhesion components have differential sensitivity to reduction in muscle contractility

To quantify the levels of integrins and IAPs at the MAS, we employed the following procedure. Two sets of embryos, aged 19–20 h after egg-laying, were imaged live within a 1 h interval. Embryos from both sets carried a single paternally provided copy of the gene encoding the fluorescently tagged protein, and one set was homozygous for *Mhc^1^*, *GluRIIC^1^* or *zip^2^*, while the other set was heterozygous for these mutations. After images were acquired, the individual MASs were identified in the z-stacks with a Matlab script that identified them as objects based on their shape, orientation and size, and then the mean intensity and area of each identified MAS were measured. The values of intensities and areas were averaged in each embryo, and the average value for the homozygous mutant was expressed relative to the average value in the heterozygous siblings (set to 100%). Fluorescently labelled microspheres were used to validate that the imaging was quantitative (electronic supplementary material, figure S1; Material and methods).

**Figure 3.**
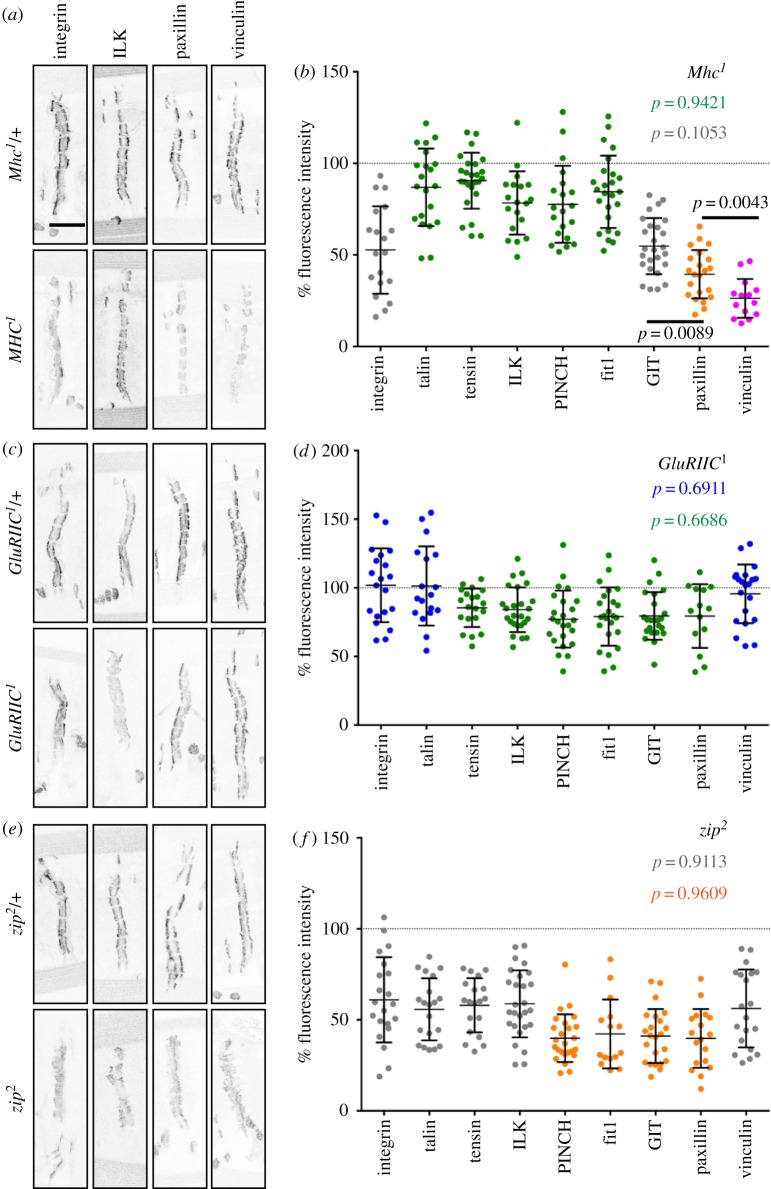
Levels of integrin adhesion components in *Mhc^1^*, *GluRIIC^1^* or *zip^2^* homozygous embryos. Examples of integrin βPS subunit (β-integrin), ILK, paxillin and vinculin localization at individual MASs in embryos homozygous and heterozygous for *Mhc^1^* (*a*), *GluRIIC^1^* (*c*) and *zip^2^* (*e*). Quantification of protein levels at MASs in embryos homozygous for *Mhc^1^* (*b*), *GluRIIC^1^* (*d*) and *zip^2^* (*f*) normalized to the mean levels in corresponding heterozygous controls. Each point represents the mean level of corresponding protein in a single embryo from one of two experimental replicates. Levels of components that are not significantly different from each other and exact *p*-values are depicted in same colours. Differences between vinculin and paxillin, and between paxillin and integrin βPS subunit and GIT are shown (*p*-values in black, *b*). In all other cases, differences between groups were *p* < 0.0001. The mean values of protein levels, sample sizes and *p*-values in comparison to heterozygous controls and *Mhc^1^* mutants are in table 2.

To confirm that contractility plays a role in the assembly of integrin adhesions at the MASs and to quantify its overall effect, we quantified the accumulation of integrin and the eight IAPs in the absence of any contractile behaviour in the muscles homozygous for *Mhc^1^*. All integrin adhesion components were still detected at MASs (figure 3*a*,*b* and table 2), in contrast with the complete disassembly of integrin adhesions in cultured cells when contractility was blocked by inhibitors [9]. The size of the adhesion structure (average area of MASs) was not affected by loss of contraction (electronic supplementary material, table S1). The levels of all proteins were reduced in comparison to the heterozygous controls, confirming that the formation of the integrin adhesion structures does respond to contractile force. The degree of the reduction differed between individual proteins (figure 3*a*,*b* and table 2), suggesting that there is more than one mechanism linking force to recruitment.

**Table 2.** The average levels (mean ± s.e.m.) of integrin adhesion components at the MASs in *Mhc^1^*, *GluRIIC^1^* and *zip^2^* homozygous embryos relative to heterozygous controls. The numbers of homozygous/heterozygous embryos that were used in each case are shown in brackets.

	*Mhc^1^*	*p*-value	*GluRIIC^1^*	*p*-value	*zip^2^*	*p*-value
β-integrin	53 ± 5*** [19/13]	<0.0001	102 ± 6 [20/14]	0.8517 (<0.0001)	61 ± 5*** [21/18]	<0.0001 (0.2759)
talin	87 ± 5* [21/15]	0.0287	101 ± 7 [18/24]	0.8855 (0.0398)	56 ± 4*** [20/19]	<0.0001 (<0.0001)
ILK	78 ± 4** [19/19]	0.0021	84 ± 3** [25/20]	0.0088 (0.2724)	59 ± 3*** [27/24]	<0.0001 (0.0004)
PINCH	78 ± 5* [20/14]	0.0119	77 ± 4** [24/23]	0.0092 (0.9510)	40 ± 3*** [25/23]	<0.0001 (<0.0001)
tensin	90 ± 3* [25/27]	0.0356	85 ± 3** [20/14]	0.0032 (0.2581)	58 ± 3*** [19/15]	<0.0001 (<0.0001)
fit1	84 ± 3* [25/20]	0.0228	79 ± 4* [24/15]	0.0103 (0.3628)	42 ± 5*** [15/12]	<0.0001 (<0.0001)
GIT	55 ± 3*** [26/25]	<0.0001	80 ± 4** [24/25]	0.0012 (<0.0001)	41 ± 3*** [23/22]	<0.0001 (0.0026)
paxillin	39 ± 3*** [22/22]	<0.0001	79 ± 6* [14/20]	0.0298 (<0.0001)	40 ± 4*** [18/21]	<0.0001 (0.9389)
vinculin	26 ± 3*** [14/19]	<0.0001	96 ± 6 [21/20]	0.6305 (<0.0001)	56 ± 5*** [20/25]	<0.0001 (<0.0001)

*p < 0.1, **p < 0.01 and ***p < 0.0001 for comparisons with corresponding heterozygous controls. The *p*-values for comparisons with levels of a particular protein in *MHC^1^* homozygous embryos are depicted in parentheses.

Vinculin and paxillin showed the strongest reduction, with vinculin being reduced more than paxillin (figure 3*a*,*b* and table 2), consistent with the greatest loss of these IAPs from focal adhesions upon inhibition of myosin II in mammalian cells by treatment with blebbistatin [29]. The βPS integrin subunit and GIT were reduced to the same degree, about 50% (figure 3*a*,*b* and table 2). Thus, muscle contraction indeed reinforces cell–ECM adhesion by increasing the accumulation of integrin receptors, in addition to reducing turnover of adhesion components [30]. The levels of the remaining five IAPs were unexpectedly reduced less than the βPS integrin subunit (*p* < 0.0001, analysis of variance (ANOVA)) to about 80% (figure 3*a*,*b* and table 2). This means that the ratio between each of these IAPs and integrin receptors increased in the absence of contractility. Therefore, the stoichiometry of integrin adhesion changes in response to muscle contractility, suggesting substantial rearrangements in interactions between the components.

Next, we examined how an intermediate reduction in contractility in *GluRIIC^1^* homozygous embryos affected adhesion components, testing whether levels of integrin and the eight IAPs were reduced in a similar way as in the *Mhc^1^* mutants, but just to a lesser extent. However, *GluRIIC^1^* homozygous embryos showed a distinct biphasic pattern of reduction, with six components of integrin adhesion reduced and three not (figure 3*c*,*d* and table 2). As with complete loss of muscle contraction, MAS area was not altered (electronic supplementary material, table S1). The three proteins that did not change were the integrin βPS subunit, talin and vinculin (figure 3*c*,*d* and table 2). Notably, these proteins are also the only ones known to undergo direct conformational changes upon applied force [14,15,31], suggesting that even the moderate amount of contractility in *GluRIIC^1^* mutants is sufficient to induce the conformational changes that lead to their further recruitment or maintenance at the MASs. The other six proteins were reduced similarly, to about 80% (figure 3*c*,*d* and table 2). Notably, reduction in four of them, ILK, PINCH, tensin and Fit1, was approximately equivalent in *GluRIIC^1^* and *Mhc^1^* mutants (figure 3*a–d* and table 2). This suggests that an increase in their accumulation at the MASs above levels that are achieved in the absence of contractility requires the high contractility provided by the bursting contractions that are absent in *GluRIIC^1^* mutants. Finally, GIT and paxillin levels were reduced less in *GluRIIC^1^* mutants than in *Mhc^1^* (table 2), suggesting that the amount of these proteins is sensitive to the strength of the contractions. To summarize, we found that there are three types of behaviour in response to these distinct perturbations of muscle contractility.

### 2.3. Adhesion components are reduced in *zip*^2^ mutants more than expected from contractility reduction

Finally, we tested accumulation of integrins and eight IAPs at MASs in *zip^2^* mutants, in which most of the contractility was retained. As with the other two mutants, there was no change in MAS area (electronic supplementary material, table S1), but the levels of all proteins were reduced in comparison to heterozygous controls (figure 3*e*,*f* and table 2). In contrast with *Mhc^1^* mutants, but similarly to *GluRIIC^1^* mutants, the reduction in protein levels in *zip^2^* mutant embryos was biphasic: five proteins were reduced to about 60%, and the other four to about 40% (figure 3*f* and table 2). Surprisingly, six out of the nine proteins were reduced more strongly than in *Mhc^1^* homozygotes (talin, ILK, PINCH, fit1, tensin, GIT; figure 3*e*,*f* and table 2). Levels of two proteins, integrin βPS subunit and paxillin, were reduced as much as in *Mhc^1^* homozygotes, and only vinculin was reduced less (figure 3*e*,*f* and table 2). Additionally, the degree of ILK and PINCH reduction differed (figure 3*f* and table 2), indicating that stoichiometry between the two proteins changed, increasing the ILK/PINCH ratio in *zip^2^* mutants. ILK and PINCH are constituents of a tripartite ILK–PINCH–parvin complex [32] and are present in a 1 : 1 : 1 ratio at MASs in wild-type animals (Y. Inoue and N.H.B. 2014, personal communication). ILK is required to recruit PINCH to MASs [33], and these results show that myosin II is needed to maintain an equimolar ratio. The excess of ILK relative to PINCH in *zip^2^* mutants raises the possibility that ILK might function independently of PINCH, which is considered its obligate partner [34].

Altogether, the changes of integrins and IAPs at MASs were the strongest in embryos homozygous for *zip^2^*, relative to the other two mutants, despite having the weakest reduction in muscle contractility. Furthermore, despite the strong reduction in the levels of all nine proteins, βPS subunit and vinculin showed the same level of reduction (figure 2*f*), which is unexpected because inhibition of non-muscle myosin II reduced vinculin more strongly than the β1 integrin subunit in migrating mammalian cells [29]. These results suggest that cytoplasmic myosin II contributes more than contractile force to the formation of MASs. It could act directly as a component of the integrin adhesion complex, contributing to MAS assembly or maintenance, as it is localized to the MAS in an integrin-dependent manner [19]. Alternatively, the reduction in integrin adhesion components could be an indirect effect of general failure of morphogenesis in *zip^2^* mutants, e.g. head involution [35].

**Figure 4.**
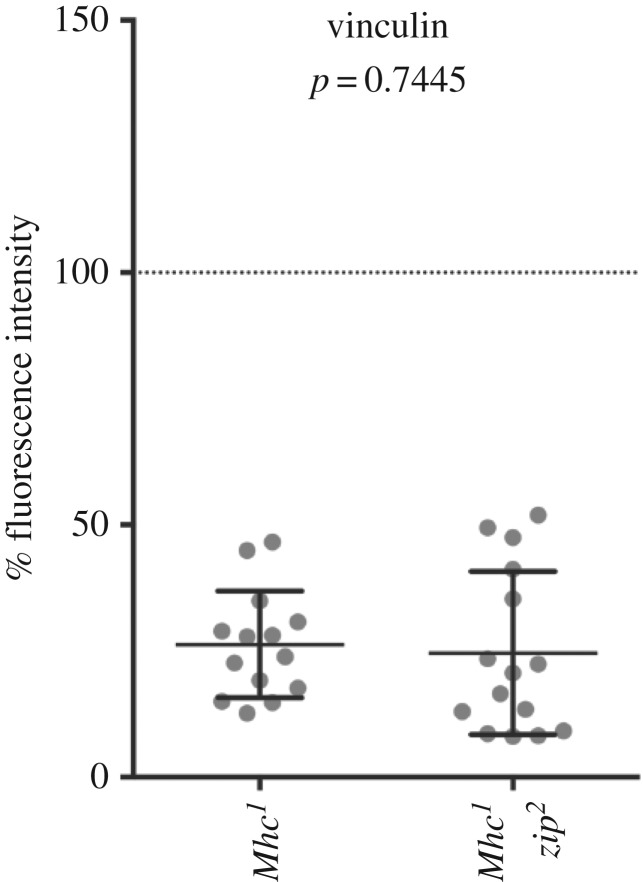
Levels of vinculin in *Mhc^1^ zip^2^* homozygous embryos in comparison to *Mhc^1^* homozygous embryos. The *p*-value corresponds to comparison between two distributions.

Finally, we tested whether the residual recruitment of the most strongly affected IAP, vinculin, to the MASs of embryos lacking muscle myosin was due to compensatory contractile activity from cytoplasmic myosin II. We generated *Mhc^1^ zip^2^* homozygous embryos lacking both myosins and found that vinculin levels were reduced to the same extent as in *Mhc^1^* homozygotes (figure 4, 26 ± 3% and 25 ± 4%, respectively). This suggests that, in a developmental context, an initial integrin adhesion site is assembled before it is subjected to the forces of myosin II contractility.

## 3. Discussion

In this work, we examined the impact of perturbing myosin activity and muscle contractions on the composition of integrin adhesion sites in *Drosophila* embryonic muscles. The first surprise was that in contrast with focal adhesions in cultured cells, which require contractile forces mediated by myosin II, none of our perturbations of myosin or contractility resulted in the absence of integrin adhesions. All of the integrin adhesion components tested were recruited to the MASs to some degree even in the complete absence of muscle contractions, similar to nascent adhesions in mammalian cells in culture, but in contrast with the rapid disassembly of integrin focal adhesions when myosin function is inhibited [9,36]. We cannot rule out the possibility that other factors within the embryo provide sufficient force on MASs to stabilize the integrin adhesions, such as the small amount of residual maternally deposited cytoplasmic myosin, actin polymerization or tissue level forces. It is also difficult to compare genetic ablation with the acute inhibition of myosin, as with genetic ablation there is potentially time for compensatory mechanisms to become established. Nonetheless, this finding is consistent with the idea that the initial muscle attachments are generated by a genetically encoded developmental programme, and then more integrins and IAPs are recruited into these adhesion structures in response to force.

**Figure 5.**
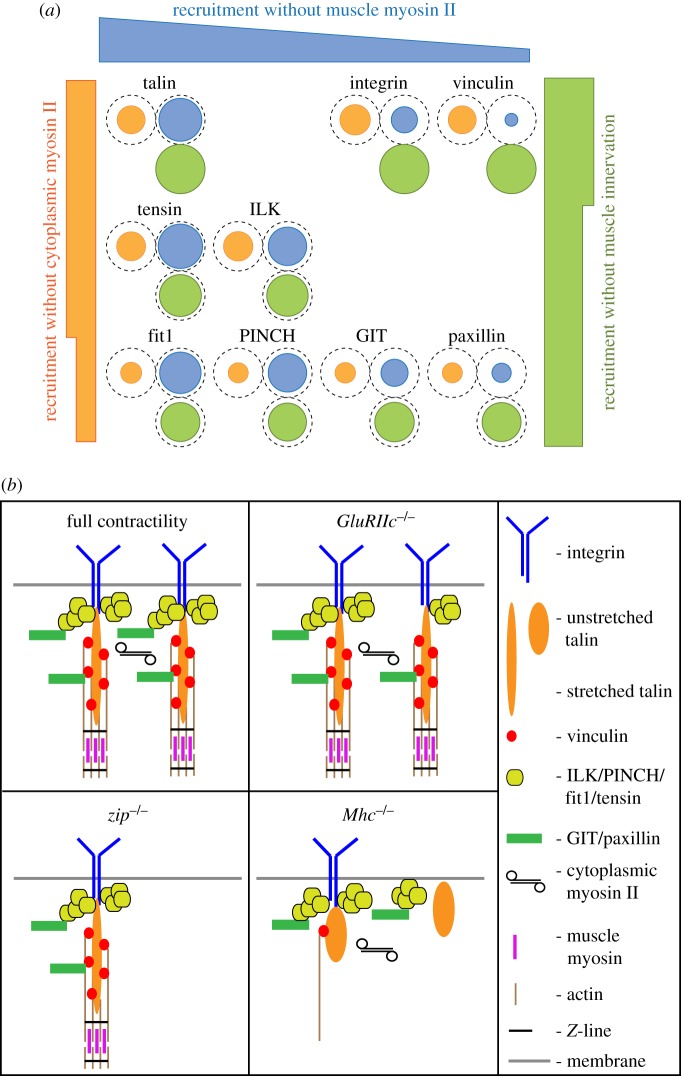
Summary of how changes to muscle contractility alter recruitment of integrin adhesion components. (*a*) Summary of protein recruitment in the three tested mutant conditions. The proteins are positioned according to their recruitment without cytoplasmic myosin II (orange, left *Y*-axis), without muscle myosin II (blue, *X*-axis) and without muscle innervation (green, right *Y*-axis). The level of recruitment is indicated by the diameter of the circles. (*b*) Simplified model of how changes in contractility affect the composition of integrin adhesions and interactions between adhesion components at muscle attachment sites. Components of integrin adhesion and connected actin cytoskeleton are depicted. In *GluRIIC^1^* mutants, integrin, talin and vinculin are recruited normally, with the rest of the components being slightly reduced. In *zip^2^* mutants, the overall reduction in recruitment of integrin adhesion components is the strongest, with cytoplasmic myosin II being a component of integrin adhesion itself, required for formation of sarcomeres, and generally involved in embryo morphogenesis. Finally, in *Mhc^1^* mutants, the protein recruitment is affected differentially, with some of the components being elevated relative to integrin in comparison to the control. This might occur through additional low-affinity interaction, for example with plasma membrane. For more details, see the text.

The second surprise for us was how differently the recruitment of each protein was affected by these three alterations to muscle contraction (summarized in figure 5). Only two pairs of proteins show equivalent behaviours (tensin and ILK, and fit1 and PINCH), giving us seven statistically distinct behaviours among the nine proteins. We might have expected proteins that we know directly interact to respond in a similar way, consistent with the subcomplexes that form in the cytoplasm [22], but this was not the case. For example, talin, tensin and fit1 can directly bind the cytoplasmic tail of integrin β subunits [37–39], and therefore might be expected to be reduced to the same degree as the integrin βPS subunit in *Mhc^1^* mutants, but instead they were reduced less. A model explaining how their ratio to integrin becomes elevated is that, in the absence of force, these IAPs make alternative interactions with MAS components, which are not strong enough to maintain them at MASs in normal, actively contracting, muscles (figure 5*b*). One candidate partner for these weaker interactions is the plasma membrane, as talin, fit1 and vertebrate tensin can all bind membrane lipids [40–42]. A shift from a high-affinity interaction with a primary binding partner to lower-affinity interactions with secondary partners in the absence of contractility is consistent with the increased dynamic exchange of integrin adhesion components in and out of adhesion sites upon reduction in contractility [30,43]. For example, a mild reduction in contractility causes a greater increase in the mobility of tensin and ILK than of integrin [30], consistent with a model where their MAS localization is maintained by new, lower-affinity interactions.

Evidence for myosin-dependent rearrangement of interactions and changes to stoichiometry arose from a study of the early recruitment of IAPs to nascent adhesions in mammalian epithelial cells [44]. The integrin α5β1, paxillin, kindlin2, talin1 and vinculin enter the nascent adhesions simultaneously. However, examination of the association between them, by cross-variance analysis, revealed that during adhesion assembly α5β1 is associated with kindlin2, and talin with vinculin, but these two subcomplexes do not associate until the nascent adhesion becomes stabilized by myosin II activity. As integrins are needed to form nascent adhesions, this finding suggests that the initial recruitment of talin to focal adhesions involves a transient interaction with integrins, directly or indirectly, and that talin remains at the nascent adhesion without maintaining a stable interaction with integrin. The lack of constant binding means that the stoichiometry of talin and integrin does not have to remain fixed, and indeed these authors found that the ratio of integrin and talin changes as nascent adhesions mature to focal adhesions. Finally, this work shows that even once the proteins are recruited to the adhesion, new interactions between them are stimulated by myosin activity.

The third surprise was that the partial reduction in contractility in *GluRIIC^1^* homozygotes did not just result in a weaker version of the effects of eliminating muscle contractions (*Mhc^1^*), but instead resulted in a different, biphasic effect. The levels of most proteins were mildly reduced, whereas levels of integrin, talin and vinculin did not change (figure 5). This was especially surprising for vinculin, given that its recruitment is the most sensitive to loss of contraction in *Mhc^1^*. This suggests that vinculin recruitment is driven by relatively low levels of force, which are already saturated prior to the advent of the stronger, innervation-dependent contractions. The reduction in ILK, PINCH, tensin and fit1 was the same in *GluRIIC^1^* and *Mhc^1^* mutant embryos, suggesting that, opposite to vinculin, the recruitment of these proteins is only stimulated by the high levels of force provided by neuronal input (figure 5). GIT and paxillin were the only IAPs whose recruitment corresponded to the degree of force in these two conditions we measured, as they were reduced in both, and more strongly in *Mhc^1^* mutants. This suggests that these proteins are recruited by force-dependent binding sites over a wide range of forces, consistent with them both having multiple potential interactors within integrin adhesions [45,46]. For example, paxillin binds both vinculin and ILK [47]. Vinculin could contribute to paxillin recruitment at low forces, and ILK could provide additional recruitment at higher forces (figure 5). To gain a clearer picture of the force dependence of protein recruitment, it would be beneficial to have a more direct measure of the forces exerted on MASs by muscle contraction. This might be achieved with force-sensing IAPs [48,49] or generating laser microlesions [50]. Force-dependent recruitment is likely to involve exposure of new binding sites, as exemplified by the stretching of talin [14]. It is also possible that the increase might be explained by changes to transcription or protein stability of the IAPs. Mechanical stimuli regulate gene expression in various systems [51], although contractility-dependent regulation of IAP expression has not, to our knowledge, been reported.

Finally, we have to explain why the ratio of PINCH and fit1 to integrin becomes higher in *Mhc^1^* mutants yet lower in *GluRIIC^1^* mutants. We explained the relative elevation of talin, tensin and fit1 in the model above by formation of weak interactions that are maintained in the absence of contractions. We can explain how the balance goes the other way if the weak inter-bursting contractions in *GluRIIC^1^* mutants are, nonetheless, sufficient to disrupt the low-affinity interactions hypothesized above. In the absence of these additional recruitment interactions, we can observe the loss of a high force-recruitment mechanism downstream of integrin.

As mentioned in the results, the best way we can explain the effects removing cytoplasmic myosin II is to hypothesize that it is an IAP that contributes to the recruitment of the IAPs that we have examined. Integrins recruit cytoplasmic myosin II to MASs [19], and minifilaments of phospho-myosin IIA have recently been found associated with focal adhesions in mammalian cells [52]. Whether this myosin provides local contractile force or has other activities remains to be discovered.

To summarize, we have described how the levels of nine components of integrin adhesion change in different contractile conditions. We found that the stoichiometry of adhesion sites depends on contractility. A model where interactions within adhesion sites are altered in response to the amount of force can explain these results, suggesting that force can produce global rearrangement of the integrin adhesion interactome. Differential accumulation of IAPs at MASs could impact on such congenital conditions as nemaline myopathy, which is caused by mutations in proteins constituting sarcomeric thin filaments [53], and myofibrillar myopathies caused by aggregation of proteins such as ZASP, filaminA and FHL1 [54]. Our findings also support the idea that MASs can be reinforced in response to exercise (e.g. [55]). Discovering the mechanisms for the changes in the levels of components of integrin adhesion in different contractile conditions, and the consequences of these changes on integrin signalling and cytoskeleton-adhesion connections are important problems for future work.

## 4. Material and methods

### 4.1. Fly stocks

All mutant alleles used are amorphs (null). To impair muscle contraction, we used *Mhc^1^*/CyO [56], *GluRIIC^1^*/CyO [57], *zip^2^*/CyO (8739, Bloomington). To measure IAP levels, we used integrin βPS subunit-GFP (insertion of GFP into the locus by homologous recombination) and GFP-tagged genomic rescue constructs: GFP-talin, vinculin-GFP (all from [58]), ILK-GFP [59], PINCH-GFP *stck^18^* [60], tensin-GFP *by^33c^* [61], Fit1-GFP [62], GIT-GFP (J. Friedlander and N.H.B. 2011, unpublished data) and paxillin-GFP [63]. All GFP-tagged genomic rescue constructs fully rescue null alleles of the corresponding gene. The flies and embryos were kept at 25°C.

### 4.2. Immunostaining and image acquisition

The embryos were collected for 1 h and allowed to develop for 19 h at 25°C. The embryos homozygous and heterozygous for *Mhc^1^*, *GluRIIC^1^* or *zip^2^* and carrying a paternal copy of GFP-tagged component of integrin adhesion were de-chorionated in 50% bleach and washed in water.

For immunostaining, embryos were heat-fixed according to the standard protocol: embryos were boiled for 30 s in 5 ml of 68 mM NaCl with 0.03% Triton X-100, immediately diluted with 15 ml of ice-cold 68 mM NaCl with 0.03% Triton X-100 and de-vitellinized for 20 s in methanol : heptane 1 : 1. Then, embryos were washed three times in methanol, and kept in methanol between 6 and 24 h at −20°, and in PBS with 0.1% Triton X-100. Rehydrated embryos were blocked for 2 h in 1% Native Goat Serum (ab7481, Abcam) in PBS with 0.1% Triton X-100. Primary antibody incubations were done overnight at 4°C. Primary antibodies used were mouse anti-GFP 1 : 250 (JL-8, Clontech), rat anti-αPS2 integrin 1 : 20 [64] and rabbit anti-obscurin 1 : 1000 [25].

For live imaging, the embryos were embedded in halocarbon oil 27 (Sigma). Individual embryos from the mixture of heterozygous and homozygous embryos were imaged in random order. The heterozygous and homozygous embryos were distinguished by the presence of Dfd::YFP carried on the balancer CyO chromosome. All measurements were performed on the attachment sites made by the dorsal muscles (figure 1*a*).

For quantification of contractility, the embryos expressing a paternal copy of GFP-talin were imaged with a combined Yokogawa CSU22 spinning disc confocal imaging system with an iXon DV855 camera (ANDOR Technology) and an Olympus IX81 inverted microscope using a 40× 1.3 NA Oil UPlanFLN objective. A single time-lapse movie was recorded from each embryo with 3–4 MASs in focus at a 0.36 s interval of 9 min in total. Images of 16-bit depth were taken at a magnification of 0.255 µm pixel^−1^. Image acquisition was done with the MetaMorph software (http://www.moleculardevices.com/Products/Software/Meta-Imaging-Series/MetaMorph.html).

For quantification of protein levels, the embryos were imaged with an Olympus FV1000 upright confocal microscope using a 60× 1.35 NA Oil UPlanSApo objective. A single stack of 14 z-sections spaced by 0.8 µm was imaged from each embryo. Images of 16-bit depth were taken at a magnification of 0.338 µm pixel^−1^. Image acquisition was done with the FV10-ASW software for Olympus FV1000. The linear relationship between the fluorescence intensity of the sample and the signal detected by the imaging system was evaluated under experimental settings using microspheres from the FocalCheck Fluorescence Microscope Test Slide system (Invitrogen, electronic supplementary material, figure S1). In all cases, the laser power was 1% to minimize the photobleaching during acquisition, scan speed was 12.5 µm s^−1^, gain was 1 and image size was 800 × 384 px. The long axis of images was manually aligned with the anterior–posterior axes of embryos. High voltage was adjusted depending on individual GFP-tagged IAP and ranged from 410 (vinculin-GFP) to 590 (Git-GFP). For all other proteins, high voltage was 510.

### 4.3. Quantification of muscle contractility

The time-lapse movies were analysed in the Fiji software (http://pacific.mpi-cbg.de/wiki/index.php/Fiji). From each movie, a kymograph was created using Multiple Kymograph plugin (http://www.embl.de/eamnet/html/body_kymograph.html). A line spanning the entire length of the image and running parallel to the embryonic anterior–posterior axis was used to create each kymograph. Durations of pauses and amplitudes of contractions during inter-burst periods were manually measured using line tool in the Fiji software. The number of bursting events during the duration of time-lapse movies was counted in each movie. The resulting measurements were exported to the R software (http://www.r-project.org/), which was used for statistical analysis. The distributions of pause durations and amplitudes of contractions during inter-bursting periods were fit using exponential distribution to estimate median pauses and amplitudes for each genotype. The numbers of bursting events per movie were fit using the Poisson distribution. The best-fit rates of the Poisson distribution were used to estimate times between bursting events using the known fixed duration of the time-lapse movies. The *p*-values for goodness of fit and the *p*-values for comparison between distributions were calculated using likelihood ratio tests.

### 4.4. Quantification of integrins and integrin-associated protein amounts at muscle attachment sites

The z-stacks were analysed using a custom-made script in the Matlab software (http://uk.mathworks.com/products/matlab/). Every frame was analysed individually as it was not possible to use z-projections due to the movement during muscle contractions. First, all objects in each frame were detected: a series of dilation, hole filling and eroding was applied to the binary image after edge detection with parameters initially manually adjusted by the investigator and then fixed throughout the analysis. The resulting objects were filtered by their area (700–4000 px), eccentricity (larger than 0.97) and orientation (more than 45° to long image axis) to exclude all objects that did not represent MASs. The above values were empirically determined from a series of tests on images of different genotypes and determining which combination was most efficient at detecting the observed MASs. Areas and mean intensities of the resulting objects were collected from original non-modified confocal frames. Then, areas and mean intensities of detected objects/MASs were averaged to produce single values for each embryo. This was done to exclude the potential for any individual embryo to make too great a contribution to the genotype average, as the number of detected objects/MASs varied widely between embryos, depending on their contractile activity during image acquisition.

The resulting values were exported to the GraphPad Prism software (http://www.graphpad.com/). The D'Agostino and Pearson normality test was used to detect deviation from the normal distribution. In all cases, the distributions of mean MAS intensities and areas passed the test. To compare the data sets, the one-way ANOVA (for three or more datasets) and unpaired *t*-test (for pair-wise comparison) were used.

## Supplementary Material

Supplementary figure and table

## Data accessibility

The datasets supporting this article have been uploaded as part of the electronic supplementary material. The Matlab script is available at https://github.com/nbul/MAS.
