# Supplementary material for: Diverse integrin adhesion stoichiometries caused by varied actomyosin activity
Source: Open Biol. 2017 Apr 26;7(4):160250. doi: 10.1098/rsob.160250 (PMC5413901; doi:10.1098/rsob.160250)
Supplement: Supplementary figure and table [file rsob160250supp1.pdf]

Table S1: The average areas (mean ± S.E.M.) of the MAS in MHC^{1}, $GluRIIC^{1}$ and zip^{2} homozygous embryos in comparison to heterozygous controls.

	Mhc^{1}	p-value	$GluRIIC^{2}$	p-value	zip^{2}	p-value
β-integrin	102 ± 5	0.8414	90 ± 3	0.0600	98 ± 5	0.6347
talin	94 ± 4	0.4162	102 ± 4	0.2157	105 ± 5	0.2128
ILK	97 ± 4	0.5736	100 ± 2	0.4138	98 ± 6	0.8791
PINCH	93 ± 3	0.2447	103 ± 5	0.6647	105 ± 4	0.1175
tensin	95 ± 3	0.1712	95 ± 3	0.3419	95 ± 3	0.3446
fit1	96 ± 4	0.4417	101 ± 4	0.8946	94 ± 4	0.3227
GIT	96 ± 4	0.4372	107 ± 4	0.2415	102 ± 4	0.7353
paxillin	108 ± 4	0.1189	99 ± 4	0.9089	109 ± 6	0.2093
vinculin	105 ± 4	0.4355	96 ± 3	0.4269	99 ± 4	0.8437

P-values (t-test) are shown for comparisons with corresponding heterozygous controls.

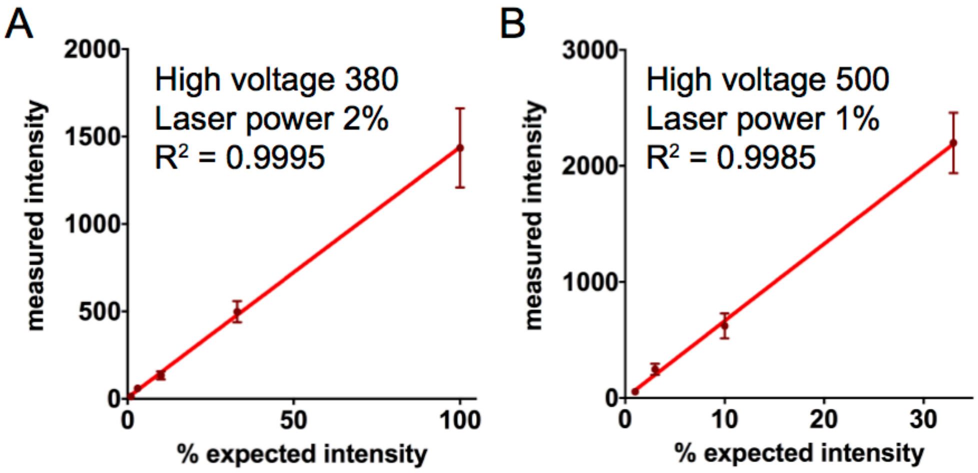

Fig.S1

Figure S1. Validation of linearity between actual amount of fluorescence and intensity measured by confocal imaging using two different experimental settings. The series of microspheres with fluorescence intensities 1%, 3%, 10%, 33% and 100% were used for image acquisition using the same fixed settings as used to detect levels of integrin adhesion components and the intensities of detected objects were quantified. The resulting intensity ± SD was plotted against the expected intensity. The goodness of fit (R^2) to linear model was quantified using GraphPad Prism software (http://www.graphpad.com/). The only parameters that differed between the two experimental settings were high voltage (380 in A and 500 in B) and laser power (2% in A and 1% in B).
